# Irritable bowel syndrome-like symptoms in treated microscopic colitis patients compared with controls: a cross-sectional study

**DOI:** 10.1093/gastro/goz069

**Published:** 2019-12-31

**Authors:** Jenny Pagoldh, David Lundgren, Ole B Suhr, Pontus Karling

**Affiliations:** Department of Public Health and Clinical Medicine, Umeå University, Umeå, Sweden; Department of Public Health and Clinical Medicine, Umeå University, Umeå, Sweden; Department of Public Health and Clinical Medicine, Umeå University, Umeå, Sweden; Department of Public Health and Clinical Medicine, Umeå University, Umeå, Sweden

**Keywords:** microscopic colitis, irritable bowel syndrome, calprotectin, anxiety, depression

## Abstract

**Background:**

The prevalence of irritable bowel syndrome (IBS)-like symptoms is high in untreated patients with microscopic colitis (MC), but there is uncertainty of the prevalence of IBS-like symptoms in treated patients. We assessed the degree of IBS-like symptoms in patients with MC in comparison to control subjects, and investigated the association between IBS-like symptoms and faecal calprotectin (FC) in MC patients.

**Methods:**

Patients with an established MC diagnosis (*n *=* *57) were compared to sex- and age-matched controls (*n *=* *138) for scores in the GSRS-IBS (Gastrointestinal Symptom Rating Scale for Irritable Bowel Syndrome) and HADS (Hospital Anxiety Depression Scale). In MC patients, an FC level was simultaneously analysed.

**Results:**

The median interval from MC diagnoses to the time the subjects participated in the study was 5.5 years (25th–75th percentiles; 4.5–9.5 years). The total GSRS-IBS score, subscores for abdominal pain, bloating, and diarrhoea were significantly higher in MC patients compared to controls (all *P *<* *0.001). There was a significant correlation between FC levels and reported bowel frequency (*P *=* *0.023), but there was no correlation between FC levels and GSRS-IBS scores. Patients with MC had significantly higher scores on anxiety (HADS-A) (*P *<* *0.001) and used more selective serotonin-reuptake-inhibitor drugs (*P *=* *0.016) than the control subjects. However, only the control subjects (not the patients with MC) showed significant correlations between GSRS-IBS scores and HADS scores.

**Conclusions:**

Patients with MC reported more IBS-like symptoms and anxiety than control subjects but neither FC levels nor symptoms of affectivity were significantly correlated with IBS-like symptoms.

## Introduction

Microscopic colitis (MC) is a chronic disorder characterized by sub-epithelial inflammation of the colonic mucosa. The concept of MC was first described in 1976 by Lindström [1]. The typical presentation of MC is recurrent watery diarrhoea and abdominal discomfort. The diagnosis of MC is based on the histological pattern of colonic biopsies that is characterized by an increase in intraepithelial lymphocytes (IEL) (>20 IEL per 100 epithelial cells) and is classified into two subtypes defined by the presence or absence of a sub-epithelial collagen layer (>10 µm) into collagenous colitis or lymphocytic colitis [2]. The incidence of MC was reported to be 25/100,000 person-years [3], to have a female predominance, and to have an increasing incidence by age [4]. Smoking, autoimmunity (i.e. rheumatic disease, thyroid disease, and coeliac disease), and some drugs (i.e. proton pump inhibitors, selective serotonin-reuptake inhibitors (SSRIs), statins, and non-steroidal anti-inflammatory drugs) have been associated with MC [5–7]; however, the cause of MC remains unclear. The most commonly used treatment for patients with MC is oral budesonide, and most patients respond to this treatment [8].

There is a symptomatic overlap between irritable bowel syndrome (IBS) and MC; several studies have demonstrated a high frequency of IBS-like symptoms in MC patients with reported prevalence of 14%–56% based on established criteria (i.e. ROME) for IBS [9–12]. However, only a few studies have compared the degree of reported gastrointestinal (GI) symptoms between MC patients and control subjects [9, 10]. In these studies, the control subjects were recruited from secondary or tertiary care and consisted of selected patients who were referred for endoscopy due to colorectal-cancer screening, with a history of anaemia or with other GI symptoms; we found no studies using control subjects that represented a normal population. Most studies performed on IBS symptoms in MC patients have focused on pre-diagnostic symptoms, and few studies have assessed GI symptoms in patients treated for MC. A recent study from Leeds in the UK reported a prevalence of 34.4% in treated patients with a new diagnosis of MC [13]. In this study, IBS-like symptoms were associated with anxiety, depression, and somatoform-type behaviour. To the best of our knowledge, the degree and characteristics of IBS-like symptoms in treated patients with MC are unknown.

The aim of this study was to estimate the degree of functional GI symptoms in patients with a treated established MC, and to compare these symptoms with a control population that represented the community. Further, we evaluated the association between IBS-like symptoms and faecal calprotectin (FC) levels in MC patients, and assessed the extent to which IBS-like symptoms were associated with symptoms of affectivity (anxiety and mood). We hypothesized that both a low-grade inflammation (measured by FC) and symptoms of affectivity have an impact on the presence of IBS-like symptoms in patients with MC.

## Materials and methods

### Study design

Cross-sectional study using questionnaires and an FC test.

### Patients with an established MC diagnosis

In 2016, all patients at Umeå University Hospital (in Västerbotten council, Sweden) who had been classified with the ICD code for MC (K52.8) between the years 2006 and 2016 were identified. A medical chart review was performed; concomitant diseases and prescriptions of medications were recorded. The criteria for MC were reviewed and only patients who met the criteria were included, i.e. >20 lymphocytes per 100 epithelial cells with or without a 10-µm sub-epithelial collagen layer in the colonic mucosa and with absence of other inflammatory bowel diseases [2]. One-hundred and nine patients met the inclusion criteria, but 11 had deceased and 1 patient had moved to another region in Sweden. This resulted in 97 patients for the study. Of these, 57 (59%) gave written consent and returned the questionnaires.

### Control subjects

**Figure 1.**
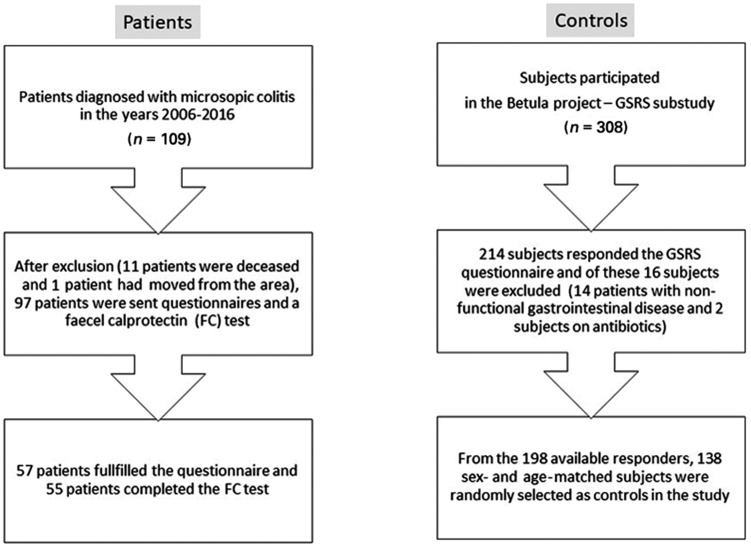
Flow chart of patients and controls recruited to the study

The control subjects were recruited from a previously described prospective study of the development of cognitive impairment in a normal population (i.e. the Betula project) [14, 15]. The subjects in that study were recruited from the same location as the MC patients. All control subjects with an established gastrointestinal disease, who had performed abdominal surgery within 3* *months before and after the survey and subjects with cognitive dysfunctions or neurological disorders were excluded [14, 15]. From the responders, control subjects for the present study were randomly selected and were sex- and age-matched (3:1) with the responders with MC. Due to differences in age and sex distribution between patients and controls, fewer controls (*n *=* *138) than originally planned were included. A flow chart for the recruitment of the patients and control subjects is presented in Figure 1.

### The Gastrointestinal Symptom Rating Scale for Irritable Bowel Syndrome (GSRS-IBS)

The Gastrointestinal Symptom Rating Scale (GSRS) is a validated self-assessment instrument used to evaluate symptoms from the gastrointestinal tract [16]. The GSRS was initially developed to be a sensitive instrument to screen for gastrointestinal symptoms in a normal population. The GSRS-IBS was a further development of the GSRS questionnaire that focused more on symptoms of IBS [17]. The GSRS uses a seven-graded Likert scale (0–6 points). In the present study, we used the GSRS-IBS and five questions from the former GSRS that included questions for gastroesophageal reflux and dyspepsia [16]. The items were clustered into symptoms groups: abdominal pain (two items), bloating (three items), constipation (two items), diarrhoea (four items), satiety (two items), gastroesophageal reflux (two items), and dyspepsia (three items) [17]. The “cluster-points” used in our study comprised the total points divided by the number of items used for each symptom-cluster. The total GSRS-IBS is displayed as the sum of all 13 GSRS-IBS points. There is no established “cut-off” for the GSRS-IBS; therefore, we used the median total GSRS-IBS score to define patients with high vs low IBS-like symptoms. In addition to the established GSRS questions, we added one question of stool frequency (“How many bowel movements did you have during the last 24 hours?”).

### The Hospital Anxiety and Depression Scale (HADS)

Symptoms of affectivity were estimated using the HADS. HADS is a highly sensitive instrument for detecting symptoms of anxiety and depression [18]. It consists of 14 questions with 7 questions focusing on anxiety (HADS-A) and 7 questions focusing on depression (HADS-D). For each question, a Likert scale (0–3) is used.

### A self-estimation of disease course

The patients with MC were also asked to select one of the four types of disease course used in the IBSEN studies [19]. The four different disease courses used in our study are “remission or mild severity of intestinal symptoms after initial high activity”, “increase in the severity of intestinal symptoms after initial low activity”, “chronic continuous symptoms”, and “chronic intermittent symptoms”.

### FC

The patients with MC mailed a stool sample with the questionnaires to the laboratory within 24 hours after performing the test. The collected samples were stored at room temperature for a maximum of 7 days before being analysed at the Laboratory of Clinical Chemistry at Umeå University Hospital. The CALPRO^®^ Calprotectin ELISA Test (ALP) was used (Calpro AS, Norway).

### Ethical consideration

The study protocol was approved by the Research Ethics Committee of Umeå University, Umeå, Sweden (Dnr 08–184 M + 07–045 M, date of approval 3 April 2007). The study protocol followed the ethical guidelines of the 1975 Declaration of Helsinki. All patients signed written consent before participating in the study.

### Statistical analysis

All statistics were calculated using SPSS version 24.0 (IBM, Armonk, NY, USA). When comparing ordinal scales and continuous variables, we used the Mann–Whitney test. The Spearman’s test was used to test correlations. The *χ*^2^ test was used for crosstabs analyses and, when the number was low (<10), the Fisher’s Exact test was used instead. The Student’s *t*-test was used for parametric comparison (age). A two-sided *P*-value <0.05 was regarded as significant. Means and standard deviations were used for continuous variables and medians and interquartiles (25th–75th percentiles) for ordinal variables. No correction for multiple testing was done.

## Results

### MC patients vs controls

The median interval from MC diagnoses to the time the subjects participated in the study was 5.5 (4.5–9.5) years. Patients with MC significantly more often had a rheumatic disease and hypothyroidism than control subjects (Table 1). In addition, concomitant use of proton pump inhibitor (PPI) therapy and SSRIs were significantly more common in MC patients (Table 1). Patients with MC reported scoring significantly higher on the total GSRS-IBS score and significantly higher for all GI symptom subscores (except for constipation) in comparison to control subjects (Table 2).

**Table 1.** Baseline characteristics of the study population

Characteristic	Patients with MC (*n* = 57)	Controls (*n* = 138)	*P*-value
Age, years, mean ± SD	60 ± 13	60 ± 13	0.964
Gender, *n* (%)			0.991
Women	43 (75)	104 (75)	
Men	14 (25)	34 (25)	
MC subtype, *n* (%)			–
Lymphocytic colitis	19 (33)	NA	
Collagen colitis	24 (42)	NA	
Unclassified MC	14 (25)	NA	
Smoking status, *n* (%)			–
Current smoker	4/41 (10)	NA	
Former smoker^a^	22/41 (54)	NA	
Never smoker	15/41 (36)	NA	
Comorbidity, *n* (%)			
Diabetes	4 (7)	5 (4)	0.452
Hypothyroidism	10 (17)	10 (7)	0.031
Rheumatic disease	4 (7)	2 (1)	0.062
Coeliac disease	6 (11)	NA	–
Concomitant therapy with, *n* (%)			
Proton pump inhibitors	15 (26)	13 (9)	0.002
NSAIDs	4 (8)	23 (17)	0.109
SSRIs/SNRIs	7 (12)	4 (3)	0.016
Statins	7 (12)	7 (6)	0.123

MC, microscopic colitis; NSAIDs, non-steroidal anti-inflammatory drugs; SSRIs, selective serotonin-reuptake inhibitors; SNRIs, serotonin- and norepinephrine-reuptake inhibitors.

^a^ Former smoker is defined as stopped smoking >1 year before the survey.

**Table 2.** Gastrointestinal symptoms and symptoms of anxiety/depression in patients with microscopic colitis (MC) and controls

Symptom	Patients with MC (*n* = 57)	Controls (*n* = 138)	*P*-value
Median GSRS- subscores (IQR)			
Abdominal pain	1.50 (2.00)	0.25 (1.50)	<0.001
Bloating	1.67 (2.33)	0.67 (1.34)	<0.001
Constipation	0 (1.50)	0 (1.00)	0.580
Diarrhoea	2.00 (2.25)	0.25 (0.75)	<0.001
Satiety	0.50 (1.00)	0 (0.50)	0.010
Reflux	0 (1.50)	0 (0.50)	0.033
Dyspepsia	0.33 (1.42)	0 (0.67)	<0.001
Median total GSRS-IBS score (IQR)	18 (18)	7 (11)	<0.001
Median HADS scores (IQR)			
Anxiety	6 (6)	3 (4)	<0.001
Depression	3 (6)	2 (3)	0.183

GSRS, Gastrointestinal Symptom Rating Scale; IQR, interquartile range; IBS, irritable bowel syndrome; HADS, Hospital Anxiety and Depression Scale.

### The use of medications for MC and IBS in patients with MC

The cumulative and current use of medications for MC and IBS are shown in Table 3. There was no difference in the median total GSRS-IBS score between patients on and not on concomitant treatment with PPI (27.0 vs 18.5; *P *=* *0.067), SSRI (18.0 vs 22.0; *P *=* *0.242), budesonide (21.5 vs 19.5; *P *=* *0.340), or any treatment for MC (budesonide, prednisolone, thiopurine, or 5-aminosalicylic acid) (25.5 vs 17.1; *P *=* *0.113). No patients had been treated with bismuth or biologics.

**Table 3.** The cumulative and current use of medications for microscopic colitis and irritable bowel syndrome in patients with microscopic colitis (*n *=* *57)

Medication	Cumulative use	Current use
Budesonide, *n* (%)	44 (77)	16 (28)
Prednisolone, *n* (%)	12 (21)	4 (7)
5-aminosalicylic acid, *n* (%)	7 (12)	1 (2)
Thiopurines, *n* (%)	3 (5)	2 (4)
Loperamide, *n* (%)	38 (67)	5 (9)
Cholestyramine, *n* (%)	22 (39)	1 (2)
Bulking agents, *n* (%)	41 (72)	5 (9)
Simethicone, *n* (%)	8 (14)	1 (2)
Spasmolytics, *n* (%)	6 (11)	1 (2)
Laxatives, *n* (%)	20 (35)	3 (5)

### Patients with high vs low GSRS-IBS scores

There were no significant differences in age, gender, smoking status, FC levels, MC type, current use, or cumulative budesonide doses, and the HADS scores between the MC patients with a high total GSRS-IBS score and patients with a low score (Table 4).

**Table 4.** Patients with microscopic colitis (MC) who reported a high score on the GSRS-IBS vs those who reported a low score (divided by the median total GSRS-IBS score)

Characteristic	Patients with high GSRS-IBS score (*n* = 30)	Patients with low GSRS-IBS score (*n* = 27)	*P*-value
Age, years, mean ± SD	58 ± 13	61 ± 13	0.338
Gender, *n* (%)			0.218
Women	25 (83)	18 (67)	
Men	5 (17)	9 (33)	
MC subtype, *n* (%)			0.755
Lymphocytic colitis	9 (30)	10 (37)	
Collagen colitis	14 (47)	10 (37)	
Unclassified MC	7 (23)	7 (26)	
Smoking status, *n* (%)			0.400
Current smoker	2/24 (8)	2/17 (12)	
Former smoker	15/24 (63)	7/17 (41)	
Never smoker	7/24 (29)	8/17 (47)	
Median faecal calprotectin, µg/g (25th–75th percentiles)	42 (25–113)	33 (25–102)	0.972
Current treatment for MC, *n* (%)	12 (41)	8 (31)	0.575
Current use of budesonide treatment, *n* (%)	8 (28)	9 (32)	0.772
Median cumulative post-diagnosis budesonide dose, mg (25th–75th percentiles)	1,140 (0–5,400)	999 (0–5,457)	0.797
Median HADS score (25th–75th percentiles)			
Anxiety	6.5 (3–10)	3 (2–7)	0.130
Depression	4 (1–8)	2 (1–4)	0.136

GSRS, Gastrointestinal Symptom Rating Scale; IBS, irritable bowel syndrome; HADS, Hospital Anxiety and Depression Scale.

### FC levels in patients with MC and the association with IBS-like symptoms

The median FC level in the patients with MC was 38 micrograms per gram faeces (µg/g). There was a correlation between the number of stools and FC levels, but no specific IBS-like symptom correlated with FC levels (Table 5). The median FC level in the patients on concomitant treatment for MC (98 vs 30 µg/g; *P *=* *0.183) and treatment with budesonide (98 vs 37 µg/g; *P *=* *0.287) was numerically higher but not significantly different from the median FC level in patients not on treatment.

**Table 5.** Correlations (Spearman’s test) between gastrointestinal symptoms and faecal calprotectin in patients with microscopic colitis (*n *=* *57)

Symptom	Faecal calprotectin	
	rs	*P*-value
GSRS-subscores		
Abdominal pain	–0.186	0.173
Bloating	–0.101	0.473
Constipation	–0.047	0.736
Diarrhoea	0.104	0.456
Satiety	–0.043	0.762
Reflux	0.009	0.946
Dyspepsia	0.000	1.000
Total GSRS-IBS score	–0.025	0.856
Bowel frequency	0.309	0.023

GSRS, Gastrointestinal Symptom Rating Scale; IBS, irritable bowel syndrome.

### Correlations between gastrointestinal symptoms and symptoms of affectivity

Patients with MC showed significantly higher scores on anxiety (HADS-A) than the control subjects, but there were no differences in scores on depression (HADS-D) between patients and controls (Table 2). However, only the control subjects (not the patients with MC) showed significant correlations between GSRS scores and HADS scores (Table 6).

**Table 6.** Correlations (Spearman’s test) between gastrointestinal symptoms and symptoms of anxiety/depression in patients with microscopic colitis and in controls

Symptom	HADS score—anxiety		HADS score—depression	
	rs (*P*-value) for patients (*n* = 57)	rs (*P*-value) for controls (*n* = 138)	rs (*P*-value) for patients (*n* = 57)	rs (*P*-value) for controls (*n* = 138)
GSRS—subscores				
Abdominal pain	0.119 (0.388)	0.372 (<0.001)	–0.018 (0.895)	0.108 (0.209)
Bloating	0.065 (0.642)	0.379 (<0.001)	–0.040 (0.775)	0.189 (0.026)
Constipation	–0.037 (0.793)	0.344 (<0.001)	–0.055 (0.694)	0.134 (0.118)
Diarrhoea	0.145 (0.295)	0.325 (0.002)	0.096 (0.489)	0.136 (0.111)
Satiety	0.014 (0.921)	0.211 (0.013)	–0.061 (0.660)	0.170 (0.046)
Reflux	0.080 (0.562)	0.080 (0.355)	–0.103 (0.454)	0.067 (0.435)
Dyspepsia	0.123 (0.373)	0.302 (<0.001)	–0.063 (0.651)	0.225 (0.008)
Total GSRS-IBS score	0.125 (0.363)	0.430 (<0.001)	0.020 (0.886)	0.190 (0.026)

GSRS, Gastrointestinal Symptom Rating Scale; IBS, irritable bowel syndrome; HADS, Hospital Anxiety and Depression Scale.

### A self-estimation of disease course

The patients with MC estimated their disease-course experience since diagnosis by selecting one of four disease courses used in the IBSEN study for inflammatory bowel disease (IBD) [19]. Thirty-six percent of MC patients reported a chronic continuous course and 26% reported a chronic intermittent course. Only 35% reported remission or mild disease after the initial disease flare. Patients with high total GSRS-IBS scores described their disease course significantly more often as chronic intermittent than the patients with low total GSRS-IBS score (48% vs 0%, *P *<* *0.001), whereas patients with low total GSRS-IBS scores described their disease course significantly more often as mild (or in remission) after the initial flare (58% vs 14%, *P *<* *0.001). The patients with a longer duration of MC (>5.5 years since diagnosis) reported a significantly higher median total GSRS-IBS score than the patients with a shorter disease duration (27.0 vs 16.0; *P *=* *0.035).

## Discussion

A higher prevalence of IBS has been reported in patients with MC in comparison to what is expected [9–12]. The aims of the present study were to investigate the prevalence of IBS-like symptoms in treated patients with MC in comparison with control subjects, and to assess the relationship between associative factors such as low-grade inflammation and affectivity. We found a higher prevalence of self-reported abdominal pain, bloating, and diarrhoea, as well as a higher total IBS score for patients with an established MC diagnosis in comparison with control subjects. The level of severity of the IBS-like symptoms in patients with MC was lower than that reported by patients with IBS [20] but was higher than those with ulcerative colitis (UC) in remission [21].

Previous studies have shown a higher prevalence of IBS/IBS-like symptoms in patients with IBD in clinical remission [22]. It has been argued that there is an association between low-grade inflammation in patients with IBD and the prevalence and severity of IBS symptoms. For example, patients with UC in remission and IBS symptoms have higher levels of cytokines [23], and symptoms of diarrhoea are correlated with FC levels in the lower spectrum of FC (<200 µg/g) [21]. In our study, we found a moderately significant correlation between the number of stools (stool frequency) and FC, but there was no significant correlation between FC levels and any other IBS-like symptoms. Therefore, factors other than inflammation seem to be involved in the pathogenesis of the residual IBS-like symptoms in patients with MC.

Symptoms of anxiety and depression were shown to be associated with a higher degree of reported IBS-like symptoms in patients with functional gastrointestinal disorders [20, 24], in patients with UC [21, 23, 25], in patients with affective disorders, and in control subjects [26]. Patients with MC use SSRI treatment more often than control subjects [7]. A recently published study showed that patients with MC who reported IBS symptoms scored significantly higher on anxiety and depression than the patients with no reported IBS symptoms [13]. In our study, the patients with a reported high score on IBS-like symptoms also reported higher scores on symptoms of affectivity, but the differences were not significant. However, surprisingly, there was no significant correlation between symptoms of affectivity and gastrointestinal symptoms in patients with MC. Therefore, factors other than affectivity seem to be involved in the pathogenesis of residual GI symptoms in patients with MC.

Visceral hypersensitivity is a common pathophysiological feature in patients with IBS [27]. We found only one small study that tested rectal sensitivity in MC patients and, contrary to patients with IBS, an increased threshold in the rectum in MC patients (compared to controls) was found [28]. Patients with MC have a high prevalence of bile-acid malabsorption [29] and it is possible that a part of the residual GI symptoms in some patients could be caused by the bile-acid malabsorption. Eosinophilic inflammation and disturbances in gut permeability or microbiota are other factors linked to MC that may cause gastrointestinal symptoms in patients with MC [30]. A possible cause of the residual GI symptoms reported by the patients with MC could be that the disease is insufficiently treated or resistant to treatment. A Spanish study showed that 25% of the patients with MC remain active with the disease after long-term follow-up [31]. In our study, 36% were still on treatment for MC. However, we found no differences in the current use or cumulative use of budesonide treatment in subjects with high vs low IBS-like symptoms. But this does not exclude that some of the MC patients with IBS-like symptoms may still benefit from more intensive treatment. Interestingly, the patients with a high degree of IBS-like symptoms had a longer MC disease duration and more frequently estimated their disease course (according to the disease-course estimates in the IBSEN study [19]) as chronic than those with a lower score of IBS symptoms.

We used GSRS questionnaires in the study. The GSRS is a sensitive instrument to screen for a diversity of GI symptoms but it is not constructed to settle a diagnosis of a functional GI disorder [16, 17]. However, the GSRS has the advantage of testing a broader spectrum of symptoms during the last week and, by utilizing a Likert scale to estimate the severity of symptoms, it was proven to be excellent for cross-sectional studies.

Most studies that have explored the comorbidity between IBD and IBS have used the ROME criteria to define IBS [22]. However, this approach has its limitations. First, the ROME criteria are primarily constructed to distinguish between organic disease and functional GI disorders [32]. Therefore, one can argue that the ROME criteria are not suitable for exploring specific functional symptoms in patients with organic disease. Second, the main ROME criteria for IBS are relatively unspecific (abdominal discomfort relieved by defecation, disturbances in stool consistency, and frequency) [32] and these criteria are suitable for many different gastrointestinal disorders [33]. Third, the ROME criteria in a cross-sectional study harbour a risk of bias, since the patient must recall symptoms that occurred several months ago—symptoms that may be caused by a flare-up of the underlying disease. This risk is reduced by using the GSRS-IBS questionnaires that only focus on the symptoms in the last week (7 days) and not for the last 3 months suggested by the ROME questionnaire. Fourth, the ROME criteria do not test the severity of the symptoms.

There are limitations to our study. We did not include colonic biopsies from the patients with MC at the time of the cross-sectional survey and there is uncertainty about whether the patients had mucosal healing. However, a clear definition of histological remission in MC has not been established [30]. In performed randomized control studies, the major outcome criterion used is “clinical response” [34]. In the few studies reporting data from colonic biopsies, a histological score reduction of 50% is often used to define treatment response [35], but virtually all patients still have at least mild lymphocyte inflammation in most colon segments despite treatment [36]. When testing for patterns of IBS in patients with MC, it is therefore a challenge to distinguish between symptoms caused by a flare of the MC disease, residual MC symptoms, and those caused by functional disturbances. To our surprise, most patients estimated their course of the disease as either chronic continuous or chronic intermittent, and only one-third seemed to have a prolonged remission.

Another limitation of the present study is the heterogeneity of medications used by the patients and it is possible that GI side effects of drugs contributed to our results. Surprisingly, the use of drugs associated with MC was still abundant among our patients (especially PPIs, SSRIs, and statins) and 37% were still on at least one of these drugs. The patients on PPIs tended to score higher on IBS score but the difference was not significant.

Although studies have shown increased FC levels in active MC [37], it has been argued that FC may not be an optimal marker for MC because neutrophils do not play a prominent role in the colon inflammation of MC and the calprotectin levels in lymphocytes are lower than in neutrophils [38]. For example, some studies have not shown increased FC levels in patients with MC [39]. Perhaps other faecal markers for inflammation such as eosinophil cationic protein and eosinophil protein X might correlate better with IBS-like symptoms in patients with MC [40].

To conclude, we confirmed an increased prevalence of IBS-like symptoms in patients with MC in comparison with a sex- and age-matched control group. However, no correlation between IBS-like symptoms and FC levels or symptoms of anxiety/depression was noted. Our findings emphasize the need to disclose the factors behind the residual MC and/or IBS-like symptoms in patients with MC.

## Authors’ contributions

J.P. participated in designing the study, collecting the data, performing statistical analyses, and interpreting the results. D.L. contributed to collecting and analysing the data and critically reviewing the final manuscript. O.B.S. participated in the analysis of the data and in writing the manuscript. P.K. participated in designing the study, collecting the data, performing statistical analyses, interpreting the results, and in writing the manuscript. All authors have read and approved the final manuscript.

## Funding

The study was supported by the Västerbotten County Council.
